# Multi-U-Net: Residual Module under Multisensory Field and Attention Mechanism Based Optimized U-Net for VHR Image Semantic Segmentation

**DOI:** 10.3390/s21051794

**Published:** 2021-03-05

**Authors:** Si Ran, Jianli Ding, Bohua Liu, Xiangyu Ge, Guolin Ma

**Affiliations:** 1 Key Laboratory of Smart City and Environment Modeling of Autonomous Region Universities, College of Resources and Environment Sciences, Xinjiang University, Urumqi 830046, China; ransi@stu.xju.edu.cn (S.R.); 107556517070@stu.xju.edu.cn (B.L.); gxy3s@stu.xju.edu.cn (X.G.); 15894636407@stu.xju.edu.cn (G.M.); 2 Key Laboratory of Oasis Ecology, Xinjiang University, Urumqi 830046, China

**Keywords:** multiscale convolutional network, VHR image, semantic segmentation, residual module under multisensory field, attention mechanism

## Abstract

As the acquisition of very high resolution (VHR) images becomes easier, the complex characteristics of VHR images pose new challenges to traditional machine learning semantic segmentation methods. As an excellent convolutional neural network (CNN) structure, U-Net does not require manual intervention, and its high-precision features are widely used in image interpretation. However, as an end-to-end fully convolutional network, U-Net has not explored enough information from the full scale, and there is still room for improvement. In this study, we constructed an effective network module: residual module under a multisensory field (RMMF) to extract multiscale features of target and an attention mechanism to optimize feature information. RMMF uses parallel convolutional layers to learn features of different scales in the network and adds shortcut connections between stacked layers to construct residual blocks, combining low-level detailed information with high-level semantic information. RMMF is universal and extensible. The convolutional layer in the U-Net network is replaced with RMMF to improve the network structure. Additionally, the multiscale convolutional network was tested using RMMF on the Gaofen-2 data set and Potsdam data sets. Experiments show that compared to other technologies, this method has better performance in airborne and spaceborne images.

## 1. Introduction

Remote sensing (RS) is a comprehensive earth observation technology developed in the 1960s, which can realize repeated detection of the same area in a short period of time. It is widely used in the fields of urban mapping [1,2,3,4], farmland management [5,6,7], military reconnaissance [8,9], and forest management [10]. However, due to the limitation of sensor resolution, it is difficult to realize high-precision VHR image mapping. With the advent of WorldView-2, Gaofen-2, and JinLin-1, and the increasing popularity of drone aerial images with centimeter-level resolution, it provides new opportunities for high-precision urban land cover classification mapping. While VHR images bring rich semantic information, they also bring new challenges to VHR image semantic segmentation. Facing the increasing trend of RS image information, there is an urgent problem regarding how to efficiently classify VHR images [11,12,13]. Therefore, this paper focuses on how to extract robust features for VHR image semantic segmentation under a complex background.

In the past few decades, pixel-based and object-oriented are two common image segmentation methods. In the pixel-based segmentation method, Charaniya [14] use a supervised parametric classification algorithm to segment aerial remote sensing images and LiDAR point clouds. Yang et al. [15] introduced spatial context features on the basis of pixel spectral features, and classified land cover based on Markov Random Field for multi-source remote sensing data. Im et al. [16] comprehensively used Artificial Immune Networks (ANNs), decision trees, and regression trees to extract urban multi-scale impervious surface information. In the object-oriented segmentation method, Secord et al. [17] extracted trees from aerial remote sensing images and LiDAR point clouds based on the object-oriented support vector machine (SVM) algorithm. Yu et al. [18] proposed a staged object-oriented segmentation method to obtain urban landscape classification information and successfully applied it to the city of Houston in Texas, USA. Benediktsson [19] combined the knowledge of morphology to capture spatial information, and a multiscale filter [20,21,22] and wavelet analysis [23,24] were used to extract spatial features from VHR images. Menart et al. [25] proposes a compact formula using the confusion statistics of a trained classifier to refine (re-estimate) the initial label hypotheses. Many of the above methods have good performance, but there are still some shortcomings. First, pixel-based segmentation methods can only reflect the spectral characteristics of a single pixel. Even if some spatial structure information is introduced, the image characteristics cannot be considered as a whole. The segmentation results often show obvious “spiced salt” phenomenon; Secondly, the object-oriented segmentation method is more dependent on the setting of the image segmentation threshold, and is easily affected by the image imaging environment and the distribution characteristics of the ground features. The two methods often need to select appropriate feature extraction and algorithms for specific objects, and it is difficult to deal with scenes with variable types of objects and different target scales.

As a subfield of machine learning, deep learning has made subversive improvements in computer vision (object detection, 3D reconstruction, three-dimensional perception, image encryption and decryption, etc. [26,27,28,29]), autonomous driving, and natural language processing, and gradually formed an end-to-end application model based on a large number of samples. CNN has also achieved surprising results in processing remote sensing images, such as scene classification and semantic segmentation [30,31,32]. As they can automatically generate powerful and representative features layer by layer in the neural network without human intervention [33], they can mine the spatial dependence between various segmented objects in the images, thus providing multiple methods for VHR image semantic segmentation [34,35,36]. Long [37] proposed a full convolutional network (FCN) for end-to-end semantic segmentation, which enables the CNN model to output low-resolution feature map; Badrinarayanan [38] improved FCN and proposed a new network structure, SegNet. The network consists of an encoder, which extracts spatial features from the image, and a decoder, which forecasts the result of the segmentation mask by sampling the feature map. Furthermore, some scholars [39] used dilated convolutions to replace the pooling layer in the CNN model, so that the model can better learn multi-scale features in the image. However, the model loses a lot of spatial details in the process of learning higher-level features. In view of Resnet’s excellent performance in image recognition, Schuegra [6] introduced a residual neural network into the U-Net network, which effectively improved multi-level feature learning ability. CNN models have gradually become the mainstream framework for semantic segmentation of high-resolution remote sensing image due to their end-to-end feature learning capability and the advantages of integrated image segmentation and pixel tagging [40].

There are still some problems to be solved in the current research based on FCNs. First, the current FCNs model generally uses convolution and pooling operations to learn the features of different levels. The filter size of the convolution layer is often fixed, resulting in the perceptual fields of neurons being confined to specific regions of the image, which is not conducive to mining the spatial context features of the image [41]. Although operations such as “dilated” convolution [42,43] can expand the neuronal field of perception with constant parameters, this operation tends to introduce sparse sampling signals, resulting in the loss of local details, which directly affects the segmentation effect, such as less effective in detecting small features in remote sensing images. We design an effective network module to learn spatial context features. Second, the mode of fusion and selection of features at different levels of the model is relatively simple, and the transferred features usually contain classification ambiguity or information unrelated to the boundary [44]. Some scholars use the conditional random field probability graph model to post-process the classification results of the FCNs model, which effectively reduces the “spiced salt” noise and improves the edges of the segmented objects, and then severely reduced the classification efficiency of the network due to the large computational effort [45,46]. Inspired by Ashish [47], we add an attention mechanism to the network to improve the learning efficiency of the model as well as the classification results. Third, the initial weight parameters of most current FCNs models are pre-trained from natural images. However, natural images are significantly different from high-resolution remote sensing images in terms of imaging conditions, shooting angles, and scene content [48], and natural images generally only contain three bands of RGB, while high-resolution remote sensing data may also contain multi-band information such as near-infrared and elevation images [49]. Comprehensive evaluation of different FCN models using the multi-band data also is valuable.

In summary, the major contributions of this work are: (1) proposing a Multi-U-Net model combining U-Net, RMMF, and an attention mechanism to extract ground objects from VHR images; (2) devising RMMF to learn multi-scale features so that global and local features can be excavated; and (3) using data sets obtained by different sensors to compare the performance of the state-of-the-art deep models.

The rest of this paper is arranged as follows: in the second part, we introduce the detailed information of our proposed Multi-U-Net; in the third part, we briefly introduce the relevant work; in the fourth part, we use RS images and aerial images to evaluate the effectiveness of our method; the results are discussed in the fifth part. Finally, conclusions are drawn in the sixth part.

## 2. Proposed Method

In this part, the concept of inception and residual is introduced to construct the residual module under multisensory field (RMMF), and the attention mechanism was used to assign different weights to each feature channel to optimize features. Afterward, an overview of the proposed Multi-U-Net is given to present a comprehensive picture. Some evaluation metrics are used to evaluate the performance of networks.

### 2.1. Residual Module under Multisensory Field

**Figure 1:**
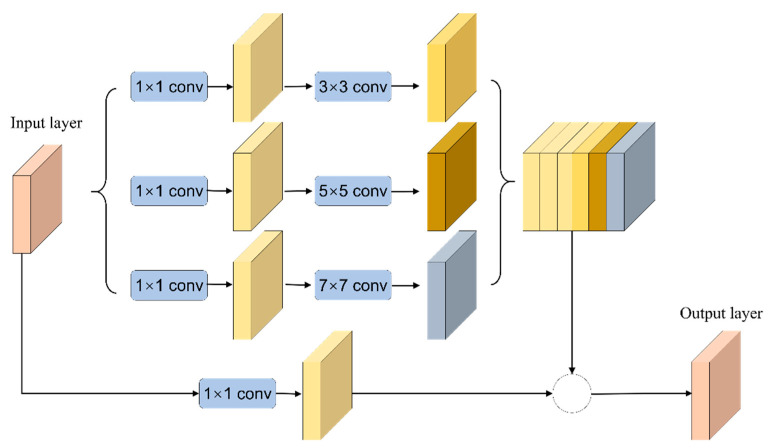
Residual module under multisensory field.

In RMMF, the inception block uses convolutional layers in parallel to learn the features of images at different scales and merges the feature information obtained from these different scales together and passes them to the next layer network. In detail, the inception block is used instead of the convolutional layer to learn the characteristics of different scales in U-Net. In the U-Net structure, two continuous 3 × 3 convolutional layers are used after each pooling layer and transposed convolutional layer. The actual meaning of these two convolution operations is similar to a 5 × 5 convolution operation, so the characteristics of the inception network are combined and expand it to 3 × 3, 5 × 5, and 7 × 7 convolution parallel operations [50]. The RMMF structure is shown in Figure 1.

The residual block is implemented in the form of skip connection, which can obtain more effective learning and rapidly reduce the training losses through a multi-layer network. The residual block takes full advantage of the identity shortcut connection and can efficiently transfer various levels of feature information between layers that are not directly connected without causing network degradation [51]. At the same time, a natural identity mapping is constructed in the module. The input and output dimensions of each neuron in the network are consistent, realizing the identification mapping of each layer input to the same layer output, and the function to be fitted in the neural network unit B(·) is split into two parts, defined as:

(1) $$z^{\left(\iota \right)}=H\left(a^{\left(\iota -1\right)}\right)\;=\;a^{\left(\iota -1\right)}\;+\;ℱ\left(a^{\left(\iota -1\right)}\right),$$

where in the feedforward neural network f(x;θ), it is composed of L nonlinear stacked units; ι∈{1≤ι≤L}; ℋ(·) is the input function; $a^{\left(\iota -1\right)}$ is the output function; ℱ(·) is the residual function; deep in the network, learning an identity map $ℋ(a^{\left(\iota -1\right)})\to a^{\left(\iota -1\right)}$ is equivalent to making the residuals approach 0, $ℱ(a^{\left(\iota -1\right)})\to 0$.

The residual block is activated after adding the input layer directly to the output layer. Therefore, the residual network can be easily implemented with the mainstream automatic differential deep learning framework and directly use the BP algorithm to update the parameters. Combining the above strategies, the residual module under multisensory field is designed, which is composed of inception and residual. The inception block uses different convolution kernels in the same layer to extract different (sparse or nonsparse) features, and the residual block is used to solve the network degradation problem.

### 2.2. Attention Mechanism

The RMMF module can make full use of the features in the network by using different convolutional layers, but there are some problems. One of the problems is that RMMF can extract multiscale features, but some of these features have a greater effect on the final semantic segmentation, while others have a lesser effect. Continuous backward transfer learning of all features will inevitably lead to errors in network training and learning, thus affecting the final result. For this reason, an attentional mechanism [47] is added in the process of backward learning that reweights the features of each channel so that the network pays more attention to the important features while suppressing the unimportant ones.

**Figure 2:**
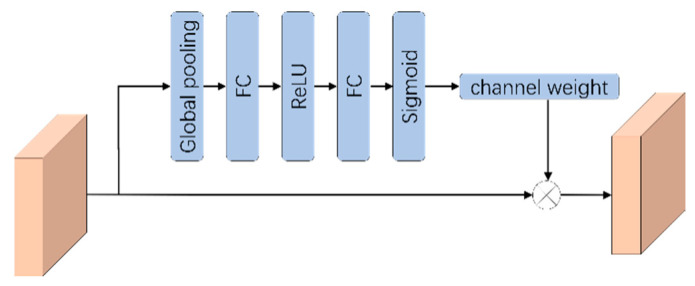
Attention mechanism.

We add the attention mechanism between the two RMMFs. That is, when the feature is passed backwards from the previous RMMF, the size of the feature graph is reduced by pooling operation, plus a module that calculates the weight of these features, reassigns the feature, and then passes these weighted features to the next RMMF. Specifically, global pooling is carried out for the output results of RMMF to obtain a 1 × 1 × C real number sequence, and then the weight of each channel feature is calculated through the full connection layer. Finally, the sigmoid function is used to normalize the weight between [0, 1]. Each channel feature is multiplied by its corresponding weight to obtain a new feature channel. The attention mechanism is shown in Figure 2.

### 2.3. Network Structure

**Figure 3:**
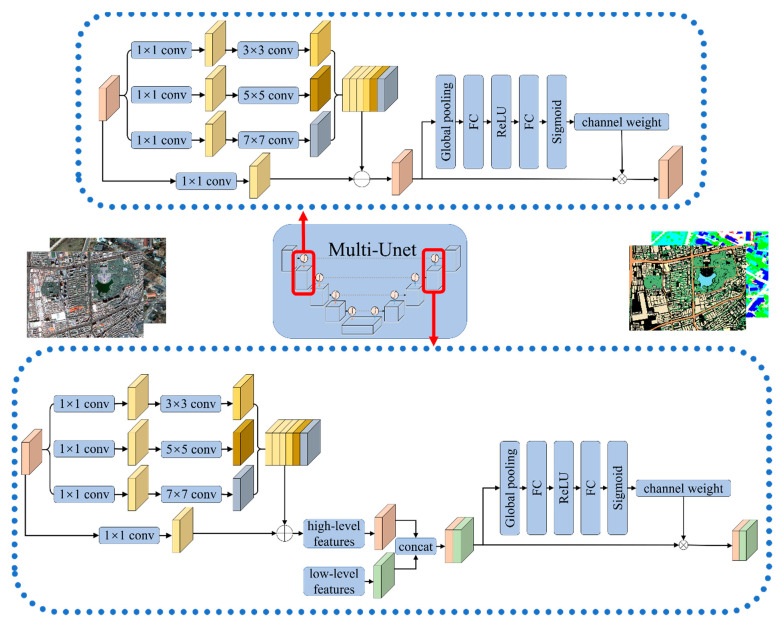
Multi-U-Net network structure.

Based on the above methods, the U-Net network is optimized and improved to obtain Multi-U-Net model (Figure 3). Multi-U-Net consists of encoder, decoder, classifier, and skip connection. The encoder part includes the RMMF, attention mechanism, and pooling layer; RMMF module by convolution kernels (3 × 3, 5 × 5, 7 × 7) expands the characteristic figure of the receptive field, namely, the feature level at each stage, studying the semantic information of images under different scales. The attention mechanism strengthens the features of important channels and weakens the features of unimportant channels to extract useful information. The pooling layer reduces the network parameters and decreases the spatial dimension gradually through continuous down-sampling to improve the robustness of image features. The decoder includes RMMF, attention mechanism, and transposed convolution layer to restore the resolution of the feature map by up-sampling the feature map. There is a fast connection between the encoder and the decoder, and the simple features in the middle and shallow layers of the network are fused with the deep abstract features to help the decoder better repair the details of the target. The attention mechanism uses the sigmoid activation function, the output layer uses the softmax activation function, and all other convolutional layers use ReLu as the activation function.

To accelerate the convergence speed of the network and prevent “gradient dispersion”, the batch normalization (BN) layer is updated in the network. For the feature map input by the entire network, each size is standardized so that the data of the feature map corresponding to the entire training sample set meets the distribution law of mean 0 and variance 1. For an input $x=\left(x^{1}\ldots x^{k}\right)$ with d dimensions:

(2) $$\mu_{\beta }=\frac{1}{m}\sum_{i=1}^{m}x_{i},$$

(3) $$\sigma_{\beta }^{2}=\frac{1}{m}\sum_{i=1}^{m}{\left(x_{i}-\mu_{\beta }\right)}^{2},$$

(4) $${\hat{x}}^{\left(k\right)}=\frac{x^{\left(k\right)}-\mu_{\beta }}{\sqrt{\sigma_{\beta }^{2}+\epsilon }\;},$$

where *m* is the batch size of the input data, $\mu_{\beta }$ represents the mean value of each dimension of the feature map, $\sigma_{\beta }^{2}$ represents the variance of each dimension of the feature map, and ϵ is the smoothing factor. To avoid the impact of feature distribution on the network learning effect, the data is normalized ${\hat{x}}_{i}$:

(5) $$y_{i}=\gamma {\hat{x}}_{i}+\beta ,$$

where in the formula, γ and β are learnable reconstruction parameters, and $y_{i}$ is the output value after the network performs batch normalization operation on the input data $x_{i}$.

## 3. Experiment Design

### 3.1. Study Area and Data Source

**Figure 4:**
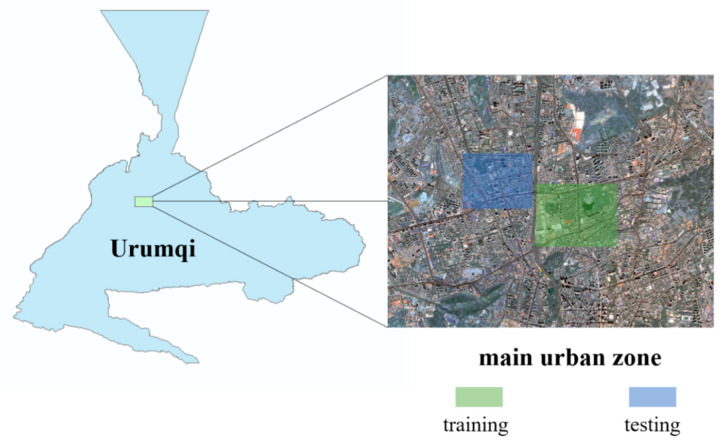
Gaofen-2 data sets.

The data set in the main urban area of Urumqi is constructed using Gaofen-2 remote sensing image. After orthorectification, geometric precision correction, atmospheric correction, fusion, and image mosaic, etc. [52], the spatial resolution reached 0.8 m, including four bands (R, G, B, NIR). According to the current status of urban land use, the labels are divided into seven types: impervious surface, building, vegetation, shadow, water, bare land, and background. As shown in the figure below (Figure 4), the green area is used as the training set, and the blue area is selected as the test sample.

Potsdam data sets (http://www2.isprs.org/commissions/comm3/wg4/2d-sem-label-potsdam.html, accessed on 22 June 2020) are public data sets provided by ISPRS-Commission III. Images were captured using digital aerial cameras by the German Association of Photogrammetry and Remote Sensing (DGPF) and mosaicked with Trimble INPHO OrthoVista. The Potsdam data set consists of 38 high resolution aerial images, covering an area of 3.42 km, and each aerial image includes four channels (R, G, B, NIR). All images are 6000 × 6000 pixels in size, including five types of tags (impervious surface, building, low vegetation, tree, and car), and the spatial resolution is 5 cm. To train and evaluate the network, five images are selected as the training set (image IDs: 03_13, 04_13, 05_13, 06_13, 07_13), and three images are selected as the test set (image IDs: 2_10, 2_11, 2_12). Due to the limitations and noise of the lidar point cloud, such as missing points and abnormal points, DSM generates some artifacts. Some buildings disappear in nDSM, and related pixels are incorrectly classified as 0, which may cause serious misclassification [40]. Therefore, nDSM is not considered in this article.

### 3.2. Comparison with Different Networks

To evaluate the effectiveness of the Multi-U-Net method, nine state-of-art FCN models, including SegNet, FCN8s, U-Net, Deeplabv3+, Inceptionv3, Res-U-Net, HSN + OI + WBP, CASIA2, and S-RA-FCN were used for comparisons. These models have been proven to be effective for semantic segmentation of remote sensing images, and they are all open source. 

SegNet, a classical encoder–decoder structure of FCNs, is often used as a baseline model to evaluate the performance of semantic segmentation. It is computationally efficient, because it reuses the positional parameters of the encoder pooling layer in the up-sampling part of the decoder, reducing the need for additional parameter training [38].

FCN8s are used by Long et al. [37] to address the problem of large loss of target edge information during segmentation. The model achieves a performance improvement of nearly 20% over the then best method on the semantic segmentation benchmark dataset PASCAL VOC2012 [53].

U-Net uses a fully convolutional network instead of a fully connected layer network for semantic segmentation [54]. It is also called the encoder–decoder structure. U-Net replicates the low-level features to the corresponding high-level features by constructing the information propagation path so that the signal can be rapidly propagated between the low-level and the high-level, which not only facilitates backward propagation during training, but can also better repair the detailed information [55,56].

Deeplabv3+ is an improved version of the third-generation model Deeplabv3 in the Deeplab series of models [57]. Compared to previous generations of models, Deeplabv3+ uses a decoder module, which further fuses the low-level features with the high-level features, thus improving the accuracy of the segmentation boundary.

Inceptionv3 uses multiple kernel filter sizes instead of stacking them sequentially so that they can be computed in parallel [58]. Compared with the previous Inception series network, through asymmetric convolutional splitting, more and richer spatial features are obtained.

Res-U-Net is a model of FCNs for semantic segmentation of buildings proposed by xu et al. [21]. The model uses ResNet-101 as an encoder and uses the Guided filters technique to post-process the classification results.

These models have been proven to be effective for semantic segmentation of remote sensing images, and they are all open source.

### 3.3. Data Processing and Method Implementation

The original image has a high resolution and is limited by hardware conditions, and inputting images directly into the model can lead to running out of memory; window sliding process was performed on the image to generate training samples and verification samples of the model. Meanwhile, to reduce the amount of calculation, divide the pixel value of each sample by 255 to scale the value to the interval [0, 1]. Data augmentation is an essential step in the task of deep learning; it generates new data by performing certain transformation operations on training data. The fundamental purpose of data augmentation is to expand the amount of data, avoid overfitting during model training, and enhance the generalization ability. We expand the training data sample size through image processing methods such as rotating, blurring, and adding noise to the sample. As a result, the training dataset contains 15,000 patches in total.

We constructed a data set of the Gaofen-2 image in Urumqi, China. At the same time, to verify the universality of the method, the Potsdam aerial image data set provided by the International Society for Photogrammetry and Remote Sensing (ISPRS) was also used. The data processing platform adopts one NVIDIA Tesla P100-PCIE-16GB GPU; the model operating environment is compiled using Keras based on the TensorFlow backend.

### 3.4. Accuracy Assessment

To evaluate the performance of network, we calculate overall accuracy (*OA*), the mean intersection over union *(*mIoU) and F1 score as evaluation indicators. *OA* is the number of correctly classified pixels as a proportion of the image data for an individual image or the entire test set; IoU refers to the ratio of correctly classified pixel numbers in a category to the sum of the ground reference pixels number and the detected pixels in the corresponding category. mIoU is the value obtained by summing up the IoU for each category and averaging them. F1 score is an “average” of both precision and recall. The calculation formula is as follows:

(6) $$F1\;=1+\beta^{2}\cdot \frac{precision\;\cdot \;recall}{\beta^{2}\;\cdot \;precision+recall},\;\beta =1$$

For each category. $precision_{k}$, $recall_{k},$ and $IoU_{k}$ are calculated as

(7) $$precision_{k}=\frac{TP_{k}}{TP_{k}+FP_{k}},recall_{k}=\frac{TP_{k}}{TP_{k}+FN_{k}},IoU_{k}=\frac{TP_{k}}{TP_{k}+FP_{k}+FN_{k}}$$

where $TP_{k}$ is the number of true positives in the category k, $FP_{k}$ is the number of false positives in the category k, and $FN_{k}$ is the number of false negatives in the category k. Furthermore, mIoU is computed by averaging all IoU scores to assess models impartially.

## 4. Experiments and Analysis

### 4.1. Comparison of the Results of the Gaofen-2 Data Set

**Figure 5:**
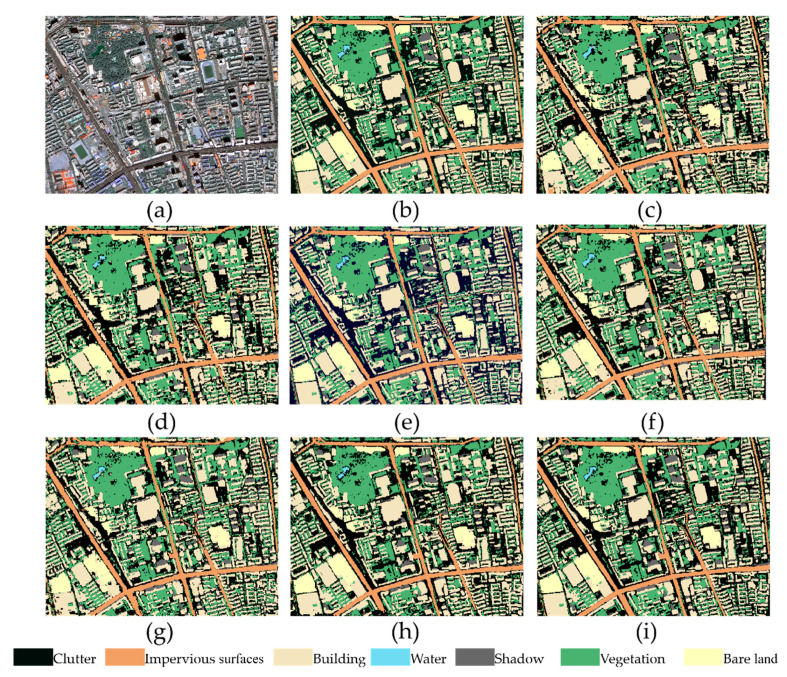
The segmentation results of different methods on the Gaofen-2 test data set. (**a**) Original image, (**b**) ground truth, (**c**) SegNet, (**d**) FCN8s, (**e**) U-Net, (**f**) Deeplabv3+, (**g**) Inceptionv3, (**h**) Res-U-Net, and (**i**) Multi-U-Net.

Semantic segmentation is performed using SegNet, FCN8s, U-Net, Deeplabv3+, Inceptionv3, Res-U-Net, and Multi-U-Net. Figure 5 shows the results of the qualitative analysis of the different methods on the Gaofen-2 data set. SegNet has obvious splicing traces in the image splicing process, and incorrectly classifies the pixels of many buildings into clutter categories. In addition, there is obvious “spiced salt” phenomenon in SegNet and FCN8s, which indicates that the upsampling operation adopted by SegNet and FCN8s cannot improve the accuracy of semantic segmentation model very well. In comparison, although U-Net also uses upsampling operations, its feature transfer and fusion functions are mainly realized by constructing the information propagation path, so its classification results are significantly better than SegNet and FCN8s. Inceptionv3 and Res-U-Net are generally better than SegNet and FCN8s in classification results, but there are also obvious errors. For example, Inceptionv3 erroneously divides bare land pixels into build pixels, while Res-U-Net has many water pixels that have not been detected. Deeplabv3+ has achieved good results on this data set, but due to shadows and vegetation occlusion, some Imp.surf. pixels are incorrectly divided. The overall classification effect of Multi-U-Net is not only the best, but it can also be seen that it is better than other models in terms of the connection degree of the impervious surfaces and the edge processing of the building (Figure 5i). This shows that RMMF can effectively improve the classification accuracy of the model by mining spatial context features.

Table 1 shows the classification accuracy of the different methods on the Gaofen-2 data set. The OA and mIoU of Multi-U-Net are 89.61% and 81.57%, which were the highest among all models. Meanwhile, Multi-U-Net has the highest classification accuracy in single category features of background, Imp.surf., building, and bare land. The overall accuracy of Res-U-Net is slightly lower than that of Multi-U-Net. The classification accuracy of vegetation and bare land is better, but the accuracy of shadows and water is lower, and there are more misclassifications; mIoU is only 79.28%. Inceptionv3 achieved the best results in the single categories of vegetation, shadows, and water, but the accuracy of bare land and build was poor. Compared with Multi-U-Net, the IoU were about 10% lower, and the OA and mIoU were 88.46% and 81.50%. Deeplabv3+ achieves better accuracy in shadows and water, with OA and mIoU of 88.63% and 81.05%.

### 4.2. Comparison of the Results of the ISPRS Potsdam Data Set

**Figure 6:**
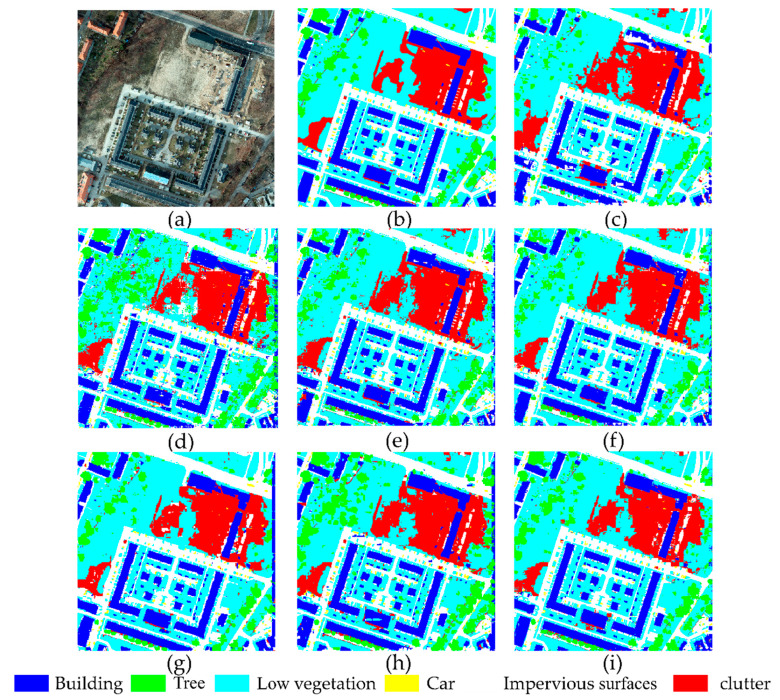
The segmentation results of different methods on the ISPRS Potsdam test data set. (**a**) Original image, (**b**) ground truth, (**c**) SegNet, (**d**) FCN8s, (**e**) U-Net, (**f**) Deeplabv3+, (**g**) Inceptionv3, (**h**) Res-U-Net, and (**i**) Multi-U-Net.

Figure 6 shows a qualitative visual comparison of different models on the Potsdam test data set. Compared with the ground reference map, Multi-U-Net has achieved satisfactory results. Generally, the more spectral information available in the data set, the higher the accuracy of the model. However, shallow models such as U-Net and SegNet tend to produce fragmented images, and the predicted targets are noisy and incoherent. Multi-U-Net with residuals obtains contextual information, which alleviates this phenomenon. Indeed, the object boundaries in our predictions are smoother and more reliable (see Figure 6). In addition, only Multi-U-Net can more completely extract white roof buildings similar to impervious surfaces in the Potsdam dataset.

Table 2 lists the qualitative results of different methods in the Potsdam test data set. The OA and mIoU of Multi-U-Net are 91.38% and 80.61%, which are better than other algorithms. At the same time, Multi-U-Net has achieved the best accuracy on impervious surface and tree. CASIA2 has the best accuracy in building extraction and car, and the overall accuracy is second only to Multi-U-Net. Inceptionv3+ and Res-U-Net perform better on the Urumqi dataset, but the mIoU on the Potsdam dataset is only 69.98% and 71.26%, indicating that the classification robustness in different scenarios needs to be improved.

### 4.3. Model Efficiency Analysis

Table 3 lists the calculated statistics of Multi-U-Net compared with other models. The model size was obtained from the model file size. The keras time command was used to compute the model train time, and the model was iterated 50 times, a total of 15,000 train samples; each sample is 256 × 256 pixels. FCN8s uses a shallow encoder architecture; it requires less computational resources and inference time than others. Deeplabv3+ has the longest reasoning time, because it uses xception block at the encoding stage. Multi-U-Net reduces the number of model parameters by using 1 × 1 convolutional kernels during the RMMF, and the model size only requires 28.34 MB, but the relatively deeper network layers increase the inference time. Overall, Multi-U-Net is more efficient than most models.

## 5. Conclusions

This paper proposes a novel model, which has the following advantages compared with other models. First of all, Multi-U-Net is an improvement of the U-Net network structure, which uses the network structure characteristics of encoder–decoder and long skip connections. Meanwhile, RMMF uses different sizes of receptive fields to mine local and global features, effectively extracts complex spatial information, and solves the problem of network training degradation through short skip connections. Secondly, the network effectively solves the problem of the transmission of redundant features in the network, and gradually optimizes feature maps of different spatial sizes. Qualitative and quantitative experimental studies on the Gaofen-2 data set and the Potsdam data set show that the method we propose can effectively improve the segmentation accuracy of urban land use and meet the feature information extraction of VHR images. In addition, the various evaluation indicators in the Potsdam data set are higher than those in the Gaofen-2 data set, which may be due to the higher spatial resolution of the Potsdam data set.

Deep learning plays an increasingly important role in the semantic segmentation of remote sensing images, and it has high efficiency in the extraction of urban land use types. In this article, we introduce a residual module under a multisensory field in the U-Net network, and by redistributing the weight of each channel feature, we propose a network structure called Multi-U-Net, which enables the network to handle semantic segmentation in VHR remote sensing images. In view of the current situation of different sizes and shapes of target objects in images, an inception block is introduced, which uses hierarchical convolution kernel to extract feature information of different scales and add residual units to the network to solve the problem of degradation caused by an excessively deep network, the attention mechanism to screen important features and weaken the unimportant features. Experiments were conducted on the Gaofen-2 data set constructed by ourselves and the public Potsdam data set. The experiments show that the performance of our proposed method is better than that of the previous method, and it has strong robustness and generalization. In general, this research provides a new method for intelligent interpretation of multi-source high-resolution remote sensing data, and explores the application of deep learning in land cover classification and typical feature information extraction. Efforts to improve the generalization ability and classification accuracy of the model is important for the implementation of territorial and spatial planning, urban disaster analysis, precision agriculture, and environmental monitoring.

## Figures and Tables

**Table 1:** The quantitative results using the deep learning models on the Gaofen-2 test data set. The bold values denote the best result, and the underlined values denote the second best result achieved by models.

Model Name	Background		Imp.surf.		Building		Vegetation		Shadow		Water		Bare Land		Overall	
	F1	IoU	F1	IoU	F1	IoU	F1	IoU	F1	IoU	F1	IoU	F1	IoU	OA	mIoU
SegNet [38]	69.83	53.65	87.13	77.20	82.20	69.78	78.07	64.03	66.75	50.09	70.65	54.62	87.61	77.95	77.17	63.90
FCN8s [37]	78.35	64.41	90.78	83.11	88.13	78.79	85.98	75.40	74.55	59.43	73.12	57.62	90.21	82.17	84.21	71.56
U-Net [54]	83.15	71.16	93.57	87.92	91.88	84.98	87.54	77.85	78.46	64.56	83.48	71.65	94.72	89.97	87.71	78.30
Deeplabv3+ [57]	83.90	72.27	93.16	87.19	91.17	83.77	90.54	82.72	83.58	71.79	90.99	83.47	92.55	86.13	88.63	81.05
Inceptionv3 [58]	83.08	71.06	91.09	83.65	87.35	77.55	**95.77**	**91.88**	**88.05**	**78.65**	**92.83**	**86.63**	89.54	81.06	88.46	81.50
Res-U-Net [21]	84.75	73.54	93.01	86.93	91.45	84.26	92.73	86.44	77.07	62.70	82.58	70.32	95.15	90.75	89.19	79.28
Multi-U-Net	**85.97**	**75.40**	**94.79**	**90.10**	**93.60**	**87.97**	88.93	80.06	80.60	67.50	87.62	77.97	**95.83**	**91.99**	**89.61**	**81.57**

**Table 2:** The quantitative results using the deep learning models on the Potsdam test data set. The bold values denote the best result, and the underlined values denote the second best result achieved by models.

Model Name	Imp.surf.		Building		Low veg.		Tree		Car		Overall	
	F1	IoU	F1	IoU	F1	IoU	F1	IoU	F1	IoU	OA	mIoU
HSN+OI+WBP [59]	91.8	--	95.7	--	84.4	--	79.6	--	88.3	--	89.4	--
CASIA2 [42]	93.3	--	**97.0**	--	**87.7**	--	88.4	--	**96.20**	--	91.1	--
S-RA-FCN [60]	91.33	--	94.70	--	86.81	--	83.47	--	94.52	--	88.59	--
SegNet [38]	89.88	81.61	81.71	69.07	56.38	39.26	86.94	76.90	71.14	55.21	84.70	64.41
FCN8s [37]	92.14	85.42	85.72	75.00	71.36	55.47	86.38	76.02	79.52	66.00	85.90	71.58
U-Net [54]	94.36	89.32	87.39	77.60	72.63	57.02	90.55	82.93	85.91	75.30	89.86	76.43
Deeplabv3+ [57]	94.58	89.72	89.26	80.61	76.80	62.34	91.55	84.41	85.37	74.47	90.94	78.31
Inceptionv3 [58]	90.57	82.77	87.61	77.96	76.00	61.28	88.98	80.15	64.61	47.73	87.49	69.98
Res-U-Net [21]	91.62	84.53	87.00	76.99	72.51	56.87	87.45	77.69	75.18	60.23	87.00	71.26
Multi-U-Net	**94.94**	**90.37**	88.63	79.58	80.66	67.58	**92.45**	**85.96**	88.62	79.56	**91.38**	**80.61**

**Table 3:** Comparisons of network efficiency among the tested deep learning models. Parameter is the number of parameters needed for model training.

Model	Parameters	Model Size (MB)	Train Time (h)
SegNet	31,821,702	121.63	4.44
FCN8s	3,050,726	11.67	2.08
U-Net	7,847,147	30.03	2.26
Deeplabv3+	41,254,646	158.63	6.13
Inceptionv3	21,815,366	84.04	2.19
Res-U-Net	110,140,324	422.32	4.21
Multi-U-Net	7,263,143	28.34	4.01

## Data Availability

Some publicly available datasets were used in this study. This data can be found here: http://www2.isprs.org/commissions/comm3/wg4/2d-sem-label-potsdam.html, accessed on 15 June 2020.
